# Thalassemia and hemoglobinopathy prevalence in a community-based sample in Sylhet, Bangladesh

**DOI:** 10.1186/s13023-023-02821-3

**Published:** 2023-07-19

**Authors:** Amanda S. Wendt, Joaquin Brintrup, Jillian L. Waid, Abdul Kader, Nathalie J. Lambrecht, Sabine Gabrysch

**Affiliations:** 1 Research Department 2, Potsdam Institute for Climate Impact Research (PIK), Member of the Leibniz Association, Potsdam, Germany; 2 Heidelberg Institute of Global Health, Heidelberg University, Heidelberg, Germany; 3 Hemoglobin Laboratory, Department of Pediatrics, University Hospital Ulm, Ulm, Germany; 4 Bangladesh Country Office, Helen Keller International, Dhaka, Bangladesh; 5 Institute of Public Health, Charité – Universitätsmedizin Berlin, Corporate Member of Freie Universität Berlin and Humboldt-Universität Zu Berlin, Berlin, Germany

**Keywords:** Hemoglobin disorder, Inherited blood disorder, Women, Children, Asia, Anemia

## Abstract

**Background:**

Inherited blood disorders affect 7% of the population worldwide, with higher prevalences in countries in the “thalassemia belt,” which includes Bangladesh. Clinical management options for severely affected individuals are expensive; thus, targeted government policies are needed to support prevention and treatment programs. In Bangladesh, there is a lack of data, in particular community-based estimates, to determine population prevalence. This study aims to estimate the prevalence of a wide range of hemoglobinopathies and their associations with anemia in a community-based sample of women and young children in rural Sylhet, Bangladesh.

**Methods:**

Capillary blood samples from 900 reproductive-aged women and 395 children (aged 6–37 months) participating in the Food and Agricultural Approaches to Reducing Malnutrition (FAARM) trial in two sub-districts of Habiganj, Sylhet Division, Bangladesh were analyzed for alpha thalassemia, beta thalassemia, and other hemoglobinopathies. We examined the association of each inherited blood disorder with hemoglobin concentration and anemia using linear and logistic regression.

**Results:**

We identified at least one inherited blood disorder in 11% of women and 10% of children. Alpha thalassemia was most prevalent, identified in 7% of women and 5% of children, followed by beta thalassemia and hemoglobin E in 2–3%. We also identified cases of hemoglobin S and hemoglobin D in this population. Having any of the identified inherited blood disorders was associated with lower hemoglobin values among non-pregnant women, largely driven by alpha and beta thalassemia. Pregnant women with beta thalassemia were also more likely to have lower hemoglobin concentrations. Among children, we found weak evidence for a relationship between hemoglobinopathy and lower hemoglobin concentrations.

**Conclusions:**

We found a high prevalence of alpha thalassemia among both women and children in rural Sylhet, Bangladesh–higher than all other identified hemoglobinopathies combined. Community-based estimates of alpha thalassemia prevalence in Bangladesh are scarce, yet our findings suggest that alpha thalassemia may comprise the majority of inherited blood disorders in some regions of the country. We recommend that future research on inherited blood disorders in Bangladesh include estimates of alpha thalassemia in their reporting for public health awareness and to facilitate couples  counseling.

**Supplementary Information:**

The online version contains supplementary material available at 10.1186/s13023-023-02821-3.

## Introduction

Inherited hemoglobin disorders affect an estimated 7% of the population worldwide and are considered the most common monogenic disease, with 300,000–500,000 infants born each year with symptomatic conditions [1]. Many more go uncounted with asymptomatic or mildly symptomatic conditions, which could worsen over time. High hemoglobinopathy prevalence is often observed in malaria-endemic regions or regions with a history of malaria. This is largely due to the protective effect of these conditions on malaria morbidity and mortality [2, 3]. This geographic area stretches from the Mediterranean to Southeast Asia and south through Sub-Saharan Africa, including Bangladesh [4, 5].

Hemoglobinopathies fall under two main categories: structural hemoglobin variants, including sickle cell – in which the structure of the hemoglobin molecule is altered – and thalassemia – in which hemoglobin synthesis (either the alpha or beta globin chain) is impaired [6]. In both alpha and beta thalassemia, when more globin chains are affected, thalassemia severity and symptoms increase. In severe cases, transfusion-dependence and iron chelation therapies are required for survival [4]. To date, there is no cure except for hematopoietic stem cell transplantation [7]. This, as well as existing treatment options, are prohibitively expensive in most low- and middle-income countries, where the majority of people with these conditions live, often leading to premature death [8].

Carriers of hemoglobin disorders may be asymptomatic or have only mild anemia and may thus be unaware of their condition. However, even silent carriers of thalassemia have been shown to exhibit altered iron metabolism and ineffective erythropoiesis, which may lead to iron overload over time due to depressed levels of hepcidin [9]. Furthermore, if two carriers have a child, there is a 25% chance that the child will suffer from a more severe condition requiring transfusion and iron chelation therapies. It is therefore of public health importance to identify those with inherited hemoglobin disorders in order to prevent ineffective and possibly detrimental interventions (e.g., iron supplementation) as well as to provide genetic counselling in case of planned pregnancies. Prevention measures such as awareness campaigns, carrier screening, and genetic counseling have been successful in reducing the burden in some settings (e.g., Cyprus, Sardinia) [10, 11]. However, in order to advocate for allocation of funding for awareness and prevention programs, information is first needed on the magnitude of this issue in a given country.

Unfortunately, data on the population prevalence of hemoglobinopathies in Bangladesh are scarce. Most reports have come from clinic-based samples which are not representative of the population and most assess only beta thalassemia and hemoglobin E [12, 13], which can be diagnosed by hemoglobin electrophoresis [14]. More comprehensive analyses to detect deletions or triplications in the alpha globin gene have rarely been reported in the literature [15]. While people with triplicated alpha globin genes do not present with symptoms, knowing the prevalence is important because, when coinherited with abnormalities on the beta globin chain, this can lead to a more severe condition due to a greater imbalance between alpha and beta globin chains [16]. No studies to date have comprehensively assessed abnormalities of both the alpha and beta globin genes, including triplicated alpha globin genes, in a community-based sample in Bangladesh. Our aim was to fill this gap by assessing a wide range of inherited blood disorders among reproductive-aged women and their young children enrolled in a nutrition-sensitive agriculture intervention trial in rural Sylhet, Bangladesh.

Estimates of inherited hemoglobin disorders could also play a role in better understanding anemia etiology in the country. In Bangladesh, reports of high anemia alongside low iron deficiency prevalence have called into question the assumption that the primary cause of anemia in this setting is iron deficiency [17–20]. Some studies have proposed that one reason for this unexplained anemia may be a higher than expected prevalence of hemoglobinopathies [17, 19, 20]. For example, one study found a 28% prevalence of beta thalassemia and hemoglobin E in a community sample of women in northwestern Bangladesh with a 57% anemia prevalence [17]. If the prevalence of inherited hemoglobin disorders is high, this would require a shift for anemia prevention and control programs in Bangladesh as iron supplementation is contraindicated in the treatment of hemoglobin disorders [21]. Universal iron supplementation programs aimed to address anemia, e.g., during antenatal care, may otherwise have unintended negative consequences. Therefore, community-based hemoglobinopathy estimates in Bangladesh are of vital importance to understand the magnitude of this issue and plan accordingly for the prevention and control of both inherited blood disorders and anemia.

## Methods

### Study population

During the baseline survey (March to May 2015) of the Food and Agricultural Approaches to Reducing Malnutrition (FAARM) trial (clinicaltrials.org: NCT02505711) in two sub-districts of Habiganj district in Sylhet Division, Bangladesh, we collected data on 2,612 women and 1,546 children. Women were recruited if they were married, their husband stayed overnight at least once in the previous year, they reported to be 30 years of age or less at enumeration, had access to at least 40 m^2^ of land, and were interested in participating in a homestead food production intervention. Children were included if they were the biological child of the enrolled woman and below 3 years of age at the start of the survey. If more than one child fit these criteria, the youngest child was included. From the full sample, a random sub-sample of the FAARM study population was selected for this study consisting of 9 households per cluster (settlement) from each of the 96 trial clusters, resulting in 934 women and their 546 children from 859 households.

**Fig. 1:**
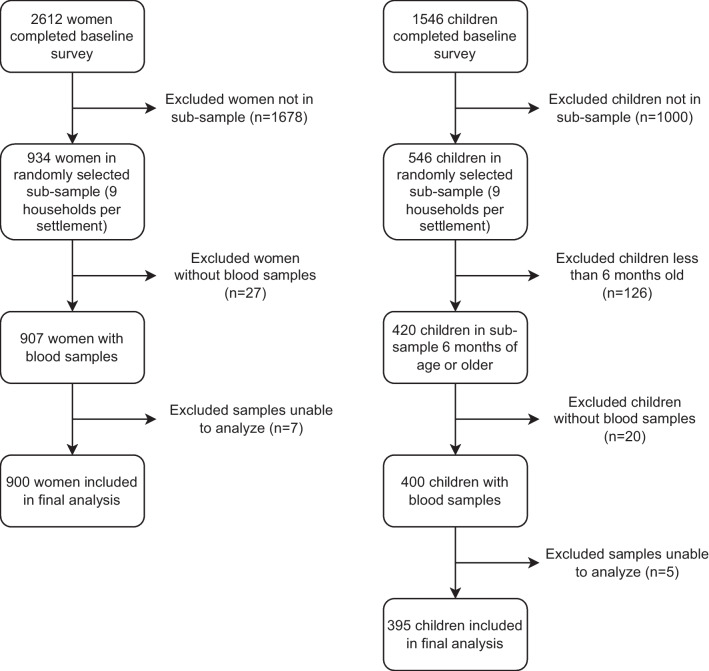
Study population for hemoglobinopathy analysis

Questionnaires were used to collect information on sociodemographic variables, including age, education (highest grade completed), household religion and wealth (as measured by a household asset index), and pregnancy duration (if applicable) with further details available in the FAARM trial protocol [22]. Capillary blood was collected from women and from children 6 months of age or older. For 7 women and 5 children, laboratory analysis to detect abnormalities in the alpha globin gene was not possible due to unknown problems such as DNA damage. This resulted in a final sample size of 900 women and 395 children, including 392 mother–child pairs (Fig. 1).

### Blood sample collection

A standard finger prick was used to collect capillary blood from women and children at the time of the survey. Hemoglobin measures were analyzed at the point of collection using Hemocue® 201 + System from the third drop of blood with standard techniques. After this, 300 µl of whole capillary blood were collected in Microvette™ tubes. Samples were immediately cooled by placing them in cool boxes with cold packs, returned to the field lab at the end of each day, and kept refrigerated overnight at 4–8 °C. The following day they were centrifuged at 12,100 x g for 10 minutes and aliquots of serum as well as the remaining pellets containing red blood cells were then stored at -20 °C. Dry ice was used during domestic and international transport to Germany. Further details on blood collection have been published in the FAARM trial protocol [22].

### Laboratory analyses

To assess beta thalassemia and structural hemoglobin variants affecting the beta globin gene, separation and quantification of hemoglobin in the stored red blood cells were performed by high-performance liquid-chromatography (HPLC) with commercial reagents and equipment (Hemoglobin Variants—HPLC, Chromsystems GmbH, Germany). The elucidation of atypical hemoglobin in the HPLC was performed by PCR amplification of the beta globin gene using specific primers and subsequent Sanger sequencing (Beckman Coulter CEQ 8800).

DNA extraction was done with the ISOLATE II Genomic DNA Kit (BIOLINE, UK) according to manufacturer instructions by LGC Lab in Berlin, Germany. Sample lysis was conducted with chaotropic salt ions and Proteinase K. Following this, genomic DNA was bound to a DNA mini spin column to wash off contaminants and impurities. The purified DNA was then eluted using an elution buffer. DNA samples were aliquoted with each aliquot normalized to a concentration of 50 µg/µl. The extracted DNA was examined for abnormalities in the alpha globin gene using Multiplex Ligation-dependent Probe Amplification (MLPA; probe mix P140-C1 HBA; MRC Holland, Amsterdam, The Netherlands). This test allowed the identification of copy number variations in the alpha globin locus, and the Hb Constant Spring point mutation in the alpha-2-globin gene.

### Variables

Women and children were defined as having “any alpha thalassemia” if they had a pathological deletion in the alpha globin locus, or if the MLPA probe Hb Constant Spring was positive. Those with triplicated alpha globin genes were identified but not included in the “any alpha thalassemia” group as this condition alone does not manifest in poor hematological outcomes [16]. Those with “any beta thalassemia” had either Hemoglobin A2 (HbA_2_) ≥ 3.5% or the sum of HbA_2_ and HbA_2_’ ≥ 3.5%.

Structural hemoglobin variants were detected via HPLC and identified by Sanger sequencing of the beta globin gene (HBB). Possible structural variants of the alpha globin genes were not identified by DNA sequencing. Individuals were classified as having “any inherited blood disorder” if either a thalassemia or structural hemoglobin variant was detected. This was the case if HPLC analysis revealed any variation in hemoglobin structure, if common mutations were identified in the beta globin gene through Sanger sequencing, or if any abnormality, excluding triplication, was found on the alpha globin gene, as detected by MLPA.

For anemia categorizations, we used the WHO recommended cut-offs for each population group (non-pregnant women: < 12 g/dL; pregnant women: < 11 g/dL for the first and third trimester, < 10.5 g/dL for the second trimester; children 6–37 months: < 11 g/dL) [23].

### Statistical analyses

All analyses were conducted using Stata/SE version 14.2. We described the prevalence of each hemoglobinopathy as well as mean hemoglobin concentration and anemia prevalence by population group: non-pregnant women, pregnant women, and children. We conducted linear regression on the relationship between hemoglobinopathy status and hemoglobin concentration, adjusting for settlement random effects, using the Stata command *mixed.* To assess the association between hemoglobinopathy status and anemia, we conducted logistic regressions adjusting for settlement random effects, using the Stata command *melogit*. For children, we additionally adjusted for age in days at time of data collection. For pregnant women, we adjusted for trimester when assessing hemoglobin concentration, while anemia cut-offs are already trimester-specific. We also assessed potential relationships between hemoglobin disorders and religion and socio-economic status using logistic regression adjusting for settlement, as described above. Regression analyses assessed alpha and beta thalassemia, hemoglobin E, and any inherited blood disorders. Other identified hemoglobinopathies were not assessed individually due to low case numbers.

## Results

Our analytic sample included 900 women of reproductive age (15–38 years old) and 395 children (6–37 months old) enrolled in the FAARM trial. Almost three quarters were from Muslim households and the remaining from Hindu households. Over 80% of women had some schooling though only 5% had completed secondary school or beyond (Table 1).

**Table 1:** Background characteristics of women and children in the FAARM study population in Sylhet, Bangladesh

Characteristics	Women		Children	
	*n* = 900		*n* = 395	
	*freq*	%	*freq*	%
Woman's age				
15–19 years	108	12.0	–	–
20–24 years	340	37.8	–	–
25–29 years	327	36.3	–	–
30–38 years	125	13 9	–	–
Child's age				
6–11 months	–	–	85	21.5
12–23 months	–	–	168	42.5
24–37 months	–	–	142	36.0
Woman's education^a^				
None	154	17.1	73	18.5
Part of primary school	194	21.6	77	19.5
Completed primary school	213	23.7	91	23.0
Part of secondary school	290	32.2	133	33.7
Completed secondary school	27	3.0	13	3.3
Higher secondary school certificate and beyond	22	2.4	8	2.0
Household religion				
Muslim	648	72.0	281	71.1
Hindu	252	28.0	114	28.9
Household wealth quintile^b^				
Poorest	213	23.7	89	22.5
Second	202	22.4	94	23.8
Third	186	20.7	71	18.0
Fourth	170	18.9	88	22.3
Richest	129	14.3	53	13.4
Anemia^c^				
Non-pregnant women/children				
None	525	68.4	200	50.6
Mild	158	20.6	108	27.3
Moderate	84	10.9	85	21.5
Severe	1	0.1	2	0.5
Pregnant women				
None	101	76.5	–	–
Mild	20	15.2	–	–
Moderate	10	7.6	–	–
Severe	1	0.8	–	–

^a^In the child column, women's education refers to the education level of the biological mother of the child

^b^Household wealth quintiles were calculated from the full FAARM population

^c^We used the following anemia cut-offs: non-pregnant women (mild: Hb 11–11.9 g/dl; moderate: 8.0–10.9 g/dL; severe: Hb < 8.0 g/dl), pregnant women in the first and third trimester (mild: Hb 10–10.9 g/dl; moderate: 7.0–9.9 g/dl; severe: Hb < 7.0 g/dl), pregnant women in the second trimester (mild: Hb 9.5–10.4 g/dl; moderate: 6.5–9.4 g/dl; severe: Hb < 6.5 g/dl), and children (mild: Hb 10–10.9 g/dl; moderate: 7.0–9.9 g/dl; severe: Hb < 7.0 g/dl)

FAARM: Food and Agricultural Approaches to Reducing Malnutrition

We identified an inherited blood disorder in 11% of women and 10% of children (Table 2). Seven individuals with an inherited blood disorder had multiple abnormalities. One woman had both alpha thalassemia trait and beta thalassemia, and three women carried the alpha thalassemia trait and also had hemoglobin E. Three children had the alpha thalassemia trait alongside another abnormality; one with beta thalassemia, one with hemoglobin E, and one with a hemoglobin mutation we were not able to identify. Having an inherited blood disorder was not associated with religion or with socio-economic status (Additional file 1: Supplementary Table 1).

**Table 2:** Thalassemia and hemoglobinopathy prevalence including genetic deletions among women and children in Sylhet, Bangladesh

Characteristics	Women		Children	
	*n* = *900*		*n* = *395*	
	*freq*	*%*	*freq*	*%*
Any inherited blood disorders	102	11.3	41	10.4
Alpha thalassemia				
Any	62	6.9	21	5.3
Minima (aa/−a)	59	6.6	21	5.3
a^37^deletion	56	6.2	19	4.8
a^42^deletion	3	0.3	2	0.5
Minor (aa/− or a−/a−)	2	0.2	0	0.0
a^37^deletions	2	0.2	0	0.0
Intermedia/Major	0	0.0	0	0.0
Hemoglobin Constant Spring	1	0.1	0	0.0
Triplicate alpha globin genes (> 5)	23	2.6	14	3.5
anti3.7_aaa_	21	2.3	14	3.5
anti4.2_aaa_	1	0.1	0	0.0
Unknown	1	0.1	0	0.0
Beta thalassemia				
Minor (HbA_2_ < 3.5%)	15	1.7	9	2.3
Intermedia/Major	0	0.0	0	0.0
Structural hemoglobin variants				
Hemoglobin E	27	3.0	7	1.8
Hemoglobin D	1	0.1	2	0.5
Hemogobin S	1	0.1	0	0.0
Hemoglobin X (unable to identify)	0	0.0	5	1.3

Categories are not mutually exlusive as some respondents had multiple inherited blood disorders: 4 women (1: αα/α-and beta thalassema; 3: αα/α-and Hemoglobin E) and 3 children (1: αα/α-and beta thalassemia; 1: αα/α-and Hemoglobin E; 1: αα/α-and an abnormality we were unable to identify)

### Alpha thalassemia

Among 7% of women and 5% of children, we identified cases of alpha thalassemia minima (-α/αα), in which one alpha globin gene is affected (almost exclusively due to the -α^3.7^ deletion) (Table 2). Two women had alpha thalassemia minor, where two alpha globin genes are not synthesized (–/αα or -α/-α), both resulting from -α^3.7^ deletions. More severe cases of alpha thalassemia intermedia or major (when three or all alpha globin chains are affected) were not identified. Hemoglobin Constant Spring, a mutation on the alpha globin gene leading to an elongation of the alpha globin chain, was identified in one woman (Table 2).

### Triplication of the alpha globin gene

In 23 women and 14 children, triplication of the alpha globin gene was identified, with most having the ααα (anti 3.7) triplication (Table 2). Alpha globin gene triplication was not considered an inherited blood disorder in our analysis as this alone does not lead to poor hematological indices. However, if an individual presents with impaired beta globin chain synthesis (i.e., beta thalassemia), alpha globin gene triplication can greatly exacerbate symptoms due to the uneven alpha globin to beta globin ratio.

### Beta thalassemia

Beta thalassemia minor, in which HbA_2_ or the sum of HbA_2_ and HbA_2_’ was 3.5% or more, was found among 2% of both women and children (Table 2). No cases of beta thalassemia intermedia or major were detected (defined as Hemoglobin F > 70%).

### Structural hemoglobin variants

We identified three structural hemoglobin variants in the study population due to point mutations on the beta globin gene (hemoglobin E, S, and D traits). We did not find severe cases of hemoglobinopathies due to homozygosity or compound heterozygosity in the beta globin gene. Hemoglobin E trait was the most common hemoglobin variant found in 3% of women and 2% of children (Table 2). Hemoglobin D trait was identified in one woman and two children. One woman was diagnosed with hemoglobin S trait. For five children, we were unable to identify the specific hemoglobinopathy. In four of these children, the beta globin gene was normal, indicating that the anomaly must be on the alpha globin gene.

### Household pairs

In around one-third of cases, if beta thalassemia was detected in a mother, it was also detected in her child (Additional file 2: Supplementary Table 2). For mothers who were silent carriers for alpha thalassemia (indicating a deletion in one out of four alpha globin loci), we found the child to be affected in approximately one-quarter of cases. For hemoglobin E, only one-fifth of children had this condition if detected in their mothers. Only one mother was identified to have Hemoglobin D, a condition which was also found in her child.

### Anemia and associations with hemoglobin disorders

Anemia was found in almost one-third of non-pregnant women (mean Hb: 12.4 g/dl), one-quarter of pregnant women (mean Hb: 11.5 g/dL), and almost half of children (mean Hb: 10.9 g/dl) (Table 3). Anemia prevalence was lower in older children (6–11 months: 73%; 12–23 months: 50%; ≥ 24 months: 34%). Among non-pregnant women, having any inherited blood disorder was strongly associated with lower hemoglobin concentrations (-0.5 g/dl) and higher odds of anemia (OR 2.1; Table 4). This was largely driven by beta thalassemia. Non-pregnant women with any beta thalassemia had 1.1 g/dl lower hemoglobin concentration and more than 7 times the odds of having anemia compared to those without any hemoglobin disorder. Non-pregnant women with alpha thalassemia also had on average 0.4 g/dl lower hemoglobin concentration than those without any hemoglobin disorder. All hemoglobinopathies together were responsible for 4% of anemia in non-pregnant women in this population, and beta thalassemia alone for 2% (population attributable fractions; Additional file 3: Supplementary Table 3).

**Table 3:** Hemoglobin concentration and anemia prevalence by thalassemia/hemoglobinopathy status among women and children in Sylhet, Bangladesh

Characteristics	Non-pregnant women			Pregnant women			Children (6–37 months)		
	*n*	Hemoglobin (g/dl)	Anemia^a^	*n*	Hemoglobin (g/dl)	Anemia^a^	*n*	Hemoglobin (g/dl)	Anemia^a^
		*Mean (SD)*	*Freq. (%)*		*Mean (SD)*	*Freq. (%)*		*Mean (SD)*	*Freq. (%)*
Total population	768	12.4 (1.2)	244 (31.7)	132	11.5 (1.2)	31 (23.5)	395	10.9 (1.3)	195 (49.4)
Any inherited blood disorders	87	12.1 (1.2)	37 (42.5)	15	10.8 (1.4)	6 (40.0)	41	10.5 (1.3)	25 (61.0)
Alpha thalassemia									
Any	53	12.2 (1.3)	21 (39.6)	9	11.2 (1.3)	3 (33.3)	21	10.8 (1.2)	10 (47.6)
Minima (aa/−a)	51	12.2 (1.3)	20 (39.2)	8	11.4 (1.3)	2 (25.0)	21	10.8 (1.2)	10 (47.6)
a^3.7^*deletion*	48	12.2 (1.3)	18 (37.5)	8	11.4 (1.3)	2 (25.0)	19	10.8 (1.2)	9 (47.4)
a^4.2^*deletion*	3	11.6 (0.8)	2 (66.7)	0	–	–	2	10.6 (1.3)	1 (50.0)
Minor (aa/− or a−/a−)									
a^3.7^*deletions*	1	11	1 (100.0)	1	9.8	1 (100.0)	0	–	–
Hemoglobin Constant Spring	1	13.3	0 (0.0)	0	–	–	0	–	–
Triplicate alpha-globin genes (> 5)	20	12.7 (1.3)	7 (35.0)	3	12.8 (0.4)	0 (0.0)	14	10.7 (1.1)	7 (50.0)
aaa^anti3.7^	18	12.7 (1.3)	6 (33.3)	3	12.8 (0.4)	0 (0.0)	14	10.7 (1.1)	7 (50.0)
aaa^anti4.2^	1	12.8	0 (0.0)	0	–	–	0	–	–
Unknown	1	11.2	1 (100.0)	0	–	–	0	–	–
Beta thalassemia									
Minor (HbA_2_ < 3.5%)	13	11.4 (1.1)	9 (69.2)	2	9.1 (0.4)	2 (100.0)	9	10.5 (1.3)	7 (77.8)
Hemoglobin E	23	12.1 (1.1)	8 (34.8)	4	10.8 (1.5)	1 (25.0)	7	10.4 (0.9)	5 (71.4)
Hemoglobin D	1	13.8 (0.0)	0 (0.0)	0	–	–	2	11.3 (0.7)	1 (50.0)
Hemoglobin S	1	13.7 (0.0)	0 (0.0)	0	–	–	0	–	–
Hemoglobin X (unable to identify)	0	0 (0.0)	0 (0.0)	0	–	–	5	9.3 (2.0)	4 (80.0)

^a^We used the following anemia cut-offs: non-pregnant women (mild: Hb 11–11.9 g/dL; moderate: 8.0–10.9 g/dL; severe: Hb < 8.0 g/dL), pregnant women in the first and third trimester (mild: Hb 10–10.9 g/dL; moderate: 7.0–9.9 g/dL; severe: Hb < 7.0 g/dL), pregnant women in the second trimester (mild: Hb 9.5–10.4 g/dL; moderate: 6.5–9.4 g/dL; severe: Hb < 6.5 g/dL), and children (mild: Hb 10–10.9 g/dL; moderate: 7.0–9.9 g/dL; severe: Hb < 7.0 g/dL)

**Table 4:** Association of inherited blood disorders with anemia and hemoglobin concentration in women and children in Sylhet, Bangladesh

Characteristics	Non-pregnant women			Pregnant women			Children^b^		
*Odds ratio (OR) for anemia*^a^	*n*	*OR (95% CI)*	*P-value*	*n*	*OR (95% CI)*	*P-value*	*n*	*OR (95% CI)*	*P-value*
Any inherited blood disorders	768	2.12 *(1.26,* 3.56)	0.004	132	2.45 (0.80, 7.54)	0.12	395	1.72 (0.80, 3.72)	0.16
Any alpha thalassemia	734	1.86 (0.98, 3.56)	0.06	126	1.86 (0.42, 8.17)	0.41	375	0.85 (0.30, 2.40)	0.76
Any beta thalassemia^c^	694	7.70 (2.02, 29.4)	0.003		***		363	4.51 (0.77, 26.5)	0.09
Hemoglobin E	704	1.33 (0.51, 3.51)	0.56	121	1.28 (0.12, 14.1)	0.84	361	2.07 (0.31, 13.9)	0.45

*Hemoglobin concentration (g/dl)*	*n*	*Beta (95% CI)*	*P-value*	*n*	*Beta (95% CI)*	*P-value*	*n*	*Beta (95% CI)*	*P-value*
Any inherited blood disorders	768	− 0.48 (− 0.74, − 0.21)	< 0.001	132	− 0.60 (− 1.23, 0.04)	0.06	395	− 0.36 (− 0.74, 0.03)	0.07
Any alpha thalassemia	734	− 0.39 (− 0.72, − 0.06)	0.02	126	− 0.40 (− 1.18, 0.38)	0.31	375	− 0.13 (− 0.64, 0.39)	0.64
Any beta thalassemia	694	− 1.09 (− 1.74, − 0.44)	< 0.001	119	− 2.10 (− 3.72, − 0.48)	0.01	363	− 0.34 (− 1.12, 0.44)	0.39
Hemoglobin E	704	− 0.43 (− 0.93, 0.06)	0.08	121	− 0.35 (− 1.54, 0.84)	0.56	361	− 0.26 (− 1.16, 0.63)	0.56

In each analysis, the reference group consists of those without any inherited blood disorder. Therefore, sample size varies between analyses. All estimates were adjusted for clustering by settlement

^a^We used the following anemia cut-offs: non-pregnant women (mild: Hb 11–11.9 g/dl; moderate: 8.0–10.9 g/dL; severe: Hb < 8.0 g/dl), pregnant women in the first and third trimester (mild: Hb 10–10.9 g/dl; moderate: 7.0–9.9 g/dl; severe: Hb < 7.0 g/dl), pregnant women in the second trimester (mild: Hb 9.5–10.4 g/dl; moderate: 6.5–9.4 g/dl; severe: Hb < 6.5 g/dl), and children (mild: Hb 10–10.9 g/dl; moderate: 7.0–9.9 g/dl; severe: Hb < 7.0 g/dl). For 11 women, pregnancy status was unknown, thus they have been excluded from this analysis as anemia cut-offs could not be calculated

^b^Child estimates were additionally adjusted for child age in days at time of blood measurement

^c^The two pregnant women with beta thalassemia were both anemic which made a regression analysis impossible

In the smaller sample of pregnant women, beta thalassemia was also associated with substantially lower hemoglobin concentration (-2.1 g/dl), but we found no evidence of an association between alpha thalassemia and hemoglobin concentration or anemia, although the direction and magnitude of effect (-0.4 g/dl) was similar to non-pregnant women (Table 4). The two pregnant women with beta thalassemia were both anemic, precluding calculation of an odds ratio.

In children, there was weak evidence that having any inherited blood disorder was associated with lower hemoglobin concentrations (-0.4 g/dl), and that having beta thalassemia was associated with anemia (OR 4.5). Beta thalassemia was responsible for 2% of anemia in children (population attributable fraction).

## Discussion

Our community-based estimate of hemoglobinopathy prevalence in Bangladesh found 11% of women and 10% of children to have an inherited blood disorder, including alpha or beta thalassemia, hemoglobin E, hemoglobin S, hemoglobin D, or other abnormalities. The most prevalent hemoglobinopathy was alpha thalassemia, which was identified in 7% of women and 5% of children. Beta thalassemia and hemoglobin E were found in 2–3% of our sample. No severe cases of thalassemia or hemoglobinopathy were identified in this community-based sample, such as alpha or beta thalassemia intermedia or major, or hemoglobin E disease. Having any inherited blood disorder was associated with lower hemoglobin concentrations and higher odds of anemia. This association was largely driven by beta thalassemia. The evidence for this relationship was very strong among non-pregnant women, though similar associations were seen in the smaller samples of pregnant women and children. To our knowledge, we are presenting the first community-based estimate in Bangladesh that includes a comprehensive analysis of both alpha and beta thalassemia as well as other hemoglobinopathies.

There is scarce literature on alpha thalassemia levels in Bangladesh to compare to our alpha thalassemia prevalence among women and children in the FAARM trial population, and existing studies report a range of estimates. One study, published in 2020, assessed alpha thalassemia in 413 newborns in three hospitals in three divisions of Bangladesh, including Sylhet, and found that 16% had at least one alpha thalassemia deletion [15]. This estimate is much higher than in our study (7% and 5%). While newborns with more severe disease may not survive to early childhood or adulthood, the older age of our child population can only explain part of the difference. In contrast, Noor et al. (2020) analyzed blood samples from 1877 young unmarried adults in Dhaka (18–35 years) from all eight divisions of Bangladesh and found only two individuals (0.1%) with alpha thalassemia trait [12]. This low alpha thalassemia prevalence (0.1%) was also found across the border in West Bengal, India in hospital-based data collected from 2005 to 2015 [24]. However, the studies in Dhaka and West Bengal used methods which target beta globin genes and did not explicitly search for abnormalities in the alpha globin genes. In general, the wide range of thalassemia prevalences found in studies from the same region is not unexpected given that genetic variation can occur even at small geographic scales. Studies in other countries, such as Thailand and Sri Lanka, have also shown large variation even between sites that are geographically very close, leading to calls for and implementation of hemoglobin disorder “micromapping” [4, 25, 26].

Interestingly, we did not identify any cases of the Southeast Asia deletion (-^SEA^), a common form of alpha thalassemia which has been reported in Bangladesh and other countries in the region [15, 27, 28]. In our study population, we found the -a^3.7^ deletion to be the most common. This finding is similar to Anwar et al. (2020), which included data from the Sylhet, Kishoreganj, and Mymensingh districts, although they also reported the Southeast Asia deletion [15]. Silent carriers of alpha thalassemia, in which only one alpha globin chain is not produced, are often asymptomatic and a lower risk of anemia is expected. However, these silent carriers may be prone to iron overload as they continue to have high iron absorption even when iron-sufficient [9]. Overall, the vast majority of the literature on inherited blood disorders in Bangladesh so far focused on beta thalassemia and hemoglobin E, while we found that the prevalence of alpha thalassemia in our population was more than the prevalence of both beta thalassemia and hemoglobin E combined. This suggests it would be important to also consider alpha thalassemia in this area.

While research on beta thalassemia has been conducted more frequently in Bangladesh, few studies examined prevalence in a community-based sample. Of two studies that did, one assessed students from classes 9 and 10 (aged 14–16 years) [13] and the other adults (aged 18–35 years) [12] living in or originally from all divisions of Bangladesh, and found similar prevalences to our findings: Khan et al. (2005) reported slightly higher prevalence among students (national: 4%; Sylhet: 5%) than Noor et al. (2020) among adults living in Dhaka (national: 2%; Sylhet: 3%). Other recent studies conducted nearby in West Bengal and eastern India also reported similar beta thalassemia prevalences (3–5%) [24, 29]. Of note, 3–4% of our study population were also found to have triplicate alpha globin genes, which if combined with beta thalassemia in offspring can exacerbate symptoms due to an increased imbalance in the alpha globin to beta globin ratio [16]. To our knowledge, this is the first community-based estimation of triplicate alpha globin genes in Bangladesh.

The prevalence of hemoglobin E in our rural Sylheti population (women: 3%; children: 2%) was slightly lower than other community-based prevalences reported in Bangladesh and the surrounding region. Khan et al. (2005) and Noor et al. (2020) reported somewhat higher prevalences in their sample of students (Sylhet: 4%; national: 6%) and adults (Sylhet: 9%; national: 9%). A study examining blood samples of hospital patients in West Bengal, India over ten years with a very large sample size (n = 119,336) and age range (5 months to 72 years) found a similar prevalence to our study (3%) [24]. One recent study in Gaibandha, Rangpur Division, Bangladesh, found a surprisingly high prevalence of hemoglobin E trait (23%) in a community sample of women without iron deficiency and 57% anemia [17]. While Merrill et al.’s sample (2012) was highly selective, a similarly high prevalence of 25% was reported by Noor et al. (2020) in a community sample of adults originally from Rangpur [12]. This likely demonstrates that the prevalence of hemoglobin disorders varies widely and can be much higher in certain areas of the country-which underlines the importance of identifying the vulnerable pockets and populations as well as continued surveillance to enable targeted public health interventions in affected areas.

When examining beta thalassemia and hemoglobin E in mother–child pairs, we found a lower than the expected 50% chance of passing each condition to one’s child (Additional file 2: Supplementary Table 2). It is possible that this may be due to early miscarriage or lower chance of survival in offspring [30]. We did not have data on inherited blood disorders of the father and so could not assess genetic inheritance from the paternal side.

Recent studies in Bangladesh have reported high anemia prevalences alongside low iron deficiency. This may point to alternative causes of anemia such as other micronutrient deficiencies (e.g., vitamin A, vitamin B12) or a potential role of inherited hemoglobin disorders. The latter were reported to be as high as 28% among women in Rangpur division in northeastern Bangladesh, driven by high levels of hemoglobin E [17]. We found overall a much lower prevalence of hemoglobinopathies in our study population in rural Sylhet (10–11%). Though we did find that inherited blood disorders were associated with lower hemoglobin concentrations in women and children, we estimate that these blood disorders were only responsible for 2–4% of anemia (Supplemental Table 3). Therefore, it is unlikely that inherited blood disorders are the primary driver for the high levels of anemia found in our population.

Our study had several strengths. The women and children included in this analysis were randomly selected from our FAARM trial population in a stratified way to ensure an even distribution over the study area. Thus, our sample was representative of our trial population and potentially other similar regions of Bangladesh. We had a large sample size and examined both young women and their children 6–37 months of age, a group which has not yet been reported on in Bangladesh. Another strength of our study is that we were able to identify thalassemia and hemoglobinopathies even in asymptomatic individuals since we did not limit our analysis to those with poor hematological indices. In addition, our chosen method (MLPA) to identify deletions/triplications in the alpha globin gene offers a substantial advantage in that it can also find previously unknown mutations-unlike other methods that can only assess previously known abnormalities.

Our study also had some limitations. Though our sample is representative of our trial population, it is not representative of Habiganj district, Sylhet division, or Bangladesh nationally. As shown by other regional studies, inherited blood disorder prevalence may vary geographically, sometimes to quite a large degree. As marital partners are often likely to be found locally versus from long distances, in addition to the varying frequency of consanguineous unions, this likely contributes to pockets of very high prevalence of inherited blood disorders in certain regions of Bangladesh. A recent study estimated 7% of marriages as consanguineous in Bangladesh, higher in rural than urban areas, with the highest estimate from rural Sylhet at 10%. At the village level, estimates ranged from 2 to 41% [31]. Further, we only collected samples from women and children, thus genetic inheritance could not be examined as we lacked paternal data. Another study limitation is that with the methods used, some beta thalassemia variants were not detectable, including delta-beta thalassemia, and beta + or beta +  + thalassemia with normal HbA_2_. These cases can only be identified by MLPA of the beta locus or sequencing of the beta globin gene of each probe, which was not feasible within this study.

## Conclusion

In our community-based sample of an apparently healthy population in rural Sylhet, Bangladesh, we found that around 10% of women and their children had a hemoglobin blood disorder, with 2% having beta thalassemia, 2–3% hemoglobin E, and 5–7% alpha thalassemia. To our knowledge, this is the first estimate of alpha thalassemia among both women and children in this region. These numbers, while elevated, likely do not explain the high levels of anemia despite lack of iron deficiency that have been reported in this region and population. The high prevalence of alpha thalassemia, which may not manifest in anemia but has been shown to result in altered iron metabolism as well as lead to more severe hemoglobin disorders of offspring, warrants attention. Further assessment should be done in the region to inform public health planning—including iron supplementation strategies—and genetic counselling services.

## Supplementary Information

**Additional file 1**. Association of inherited blood disorders with religion and wealth in women and children in Sylhet, Bangladesh.

**Additional file 2**. Mother-child pairs and prevalence of an inherited blood disorder in Sylhet, Bangladesh.

**Additional file 3**. Population attributable fractions for anemia by inherited blood disorders among women and children in Sylhet, Bangladesh.

## Data Availability

A deidentified dataset with the individual participant data that underlie the results reported in this article are available to interested researchers who provide a methodologically sound proposal for use of the data. Data requests with a proposal should be directed to the corresponding author (ASW; amanda.wendt@pik-potsdam.de) and the principal investigator (SG; sabine.gabrysch@charite.de). A data access agreement will need to be signed to gain access to the data. The FAARM trial protocol is available online.
