# Supplementary material for: Thalassemia and hemoglobinopathy prevalence in a community-based sample in Sylhet, Bangladesh
Source: Orphanet J Rare Dis. 2023 Jul 19;18:192. doi: 10.1186/s13023-023-02821-3 (PMC10355052; doi:10.1186/s13023-023-02821-3)
Supplement: Supplementary file 1 — Additional file 1. Association of inherited blood disorders with religion and wealth in women and children in Sylhet, Bangladesh. [file 13023_2023_2821_MOESM1_ESM.pdf]

Supplementary Table 1: Association of inherited blood disorders with religion and wealth in women and children in Sylhet, Bangladesh				
**Characteristics**	**Women** *(n=900)*		**Children** *(n=395)*	
Odds ratio (OR) for religion [a]	*OR (95% CI)*	*p-value*	*OR (95% CI)*	*p-value*
**Any inherited blood disorders**	0.86 (0.51, 1.46)	0.58	1.59 (0.74, 3.40)	0.24
**Any alpha thalassemia**	0.94 (0.49, 1.78)	0.84	1.25 (0.47, 3.37)	0.66
**Any beta thalassemia**	1.29 (0.44, 3.81)	0.64	3.18 (0.84, 12.1)	0.09
**Hemoglobin E**	0.73 (0.29, 1.83)	0.50	1.00 (0.14, 6.96)	1.00
Odds ratio (OR) for wealth	*OR (95% CI)*	*p-value*	*OR (95% CI)*	*p-value*
**Any inherited blood disorders**				
Wealth quintile				
Poorest	Ref	-	Ref	-
Second	0.73 (0.38, 1.41)	0.35	0.85 (0.33, 2.19)	0.73
Third	1.04 (0.55, 1.96)	0.90	1.03 (0.39, 2.77)	0.95
Fourth	0.91 (0.46, 1.77)	0.77	0.60 (0.21, 1.70)	0.34
Richest	1.32 (0.68, 2.59)	0.41	0.57 (0.16, 1.98)	0.37
**Any alpha thalassemia**				
Wealth quintile				
Poorest	Ref	-	Ref	-
Second	0.66 (0.27, 1.58)	0.35	0.67 (0.20, 2.25)	0.51
Third	1.09 (0.49, 2.43)	0.83	0.51 (0.12, 2.10)	0.35
Fourth	1.27 (0.57, 2.81)	0.56	0.70 (0.21, 2.38)	0.57
Richest	1.49 (0.65, 3.42)	0.35	0.22 (0.03, 1.92)	0.17
**Any beta thalassemia**				
Wealth quintile				
Poorest	Ref	-	Ref	-
Second	0.79 (0.17, 3.56)	0.76	0.62 (0.10, 3.82)	0.61
Third	1.44 (0.38, 5.46)	0.59	0.83 (0.14, 5.11)	0.84
Fourth	0.31 (0.03, 2.79)	0.30	0.33 (0.03, 3.23)	0.34
Richest	0.82 (0.15, 4.56)	0.82	0.55 (0.06, 5.44)	0.61
**Hemoglobin E**				
Wealth quintile				
Poorest	Ref	-	Ref	-
Second	0.90 (0.30, 2.73)	0.85	1.96 (0.16, 24.5)	0.60
Third	0.81 (0.25, 2.61)	0.73	1.24 (0.07, 23.1)	0.89
Fourth	0.71 (0.20, 2.46)	0.59	1.00 (0.05, 18.3)	1.00
Richest	1.19 (0.37, 3.82)	0.77	3.07 (0.22, 42.6)	0.40

[a]Religion defined as Hindu with reference group as Muslim
