# Supplementary material for: Thalassemia and hemoglobinopathy prevalence in a community-based sample in Sylhet, Bangladesh
Source: Orphanet J Rare Dis. 2023 Jul 19;18:192. doi: 10.1186/s13023-023-02821-3 (PMC10355052; doi:10.1186/s13023-023-02821-3)
Supplement: Supplementary file 2 — Additional file 2. Mother-child pairs and prevalence of an inherited blood disorder in Sylhet, Bangladesh. [file 13023_2023_2821_MOESM2_ESM.pdf]

Supplementary table 2: Mother-child pairs and prevalence of an inherited blood disorder in Sylhet, Bangladesh (392 pairs)

	Inherited blood disorder detected in:				
	Both mother and child	*Only mother (not child)*	*Only child (not mother)*	*Child if detected in mother (%)*	
	freq	freq	freq	%	*95% CI*
Any alpha thalassemia	9	21	12	30%	(0.15, 0.49)
Any beta thalassemia	3	5	6	38%	(0.09, 0.76)
Hemoglobin E	3	11	4	21%	(0.05, 0.51)
Hemoglobin D	1	0	1	100%	(0.03, 1.00)
Hemoglobin S	0	0	0	-	-
Any inherited blood disorder	**17**	**32**	**24**	35%	(0.22, 0.50)

CI: Confidence intervals
