# Supplementary material for: Thalassemia and hemoglobinopathy prevalence in a community-based sample in Sylhet, Bangladesh
Source: Orphanet J Rare Dis. 2023 Jul 19;18:192. doi: 10.1186/s13023-023-02821-3 (PMC10355052; doi:10.1186/s13023-023-02821-3)
Supplement: Supplementary file 3 — Additional file 3. Population attributable fractions for anemia by inherited blood disorders among women and children in Sylhet, Bangladesh. [file 13023_2023_2821_MOESM3_ESM.pdf]

Supplementary table 3: Population attributable fractions for anemia by inherited blood disorders among women and children in Sylhet, Bangladesh

	Non-pregnant women			*Pregnant women*			*Children*		
	n	*PAF*	*95% CI*	*n*	*PAF*	*95% CI*	*n*	*PAF*	*95% CI*
Any inherited blood disorder	768	0.04	(0.01, 0.08)	132	0.09	(-0.05, 0.21)	395	0.03	(-0.01, 0.06)
Any alpha thalassemia	734	0.02	(-0.01, 0.05)	126	0.04	(-0.07, 0.14)	375	-0.0005	(-0.03, 0.02)
Any beta thalassemia	694	0.02	(0.01, 0.04)	117	-	-	363	0.02	(0.00, 0.03)
Hemoglobin E	704	0.005	(-0.02, 0.03)	121	0.01	(-0.06, 0.07)	361	0.01	(-0.01, 0.02)

PAF: Population attributable fraction
